# Proactive Risk Assessment Through Failure Mode and Effect Analysis (FMEA) for Haemodialysis Facilities: A Pilot Project

**DOI:** 10.3389/fpubh.2022.823680

**Published:** 2022-03-24

**Authors:** Raffaele La Russa, Valentina Fazio, Michela Ferrara, Nicola Di Fazio, Rocco Valerio Viola, Gianluca Piras, Giuseppe Ciano, Fausta Micheletta, Paola Frati

**Affiliations:** ^1^Department of Clinical and Experimental Medicine, Section of Forensic Pathology, University of Foggia, Ospedale Colonnello D'Avanzo, Foggia, Italy; ^2^Department of Anatomical, Histological, Forensic Medicine and Orthopedic Sciences, Sapienza University of Rome, Rome, Italy; ^3^Polo Sanitario San Feliciano, Rome, Italy

**Keywords:** FMEA, haemodialysis, CVC, risk management, failure mode

## Abstract

Haemodialysis (HD) is one of the methods for renal replacement therapy in the management of advanced chronic kidney disease through an osmosis process that allows purification of blood in the dialysis machine. The complexity of the dialytic procedure often requires the presence of a multi-specialist, multi-disciplinary team. The dialysis process is an important target for clinical risk management. Failure Mode and Effect Analysis (FMEA) is a proactive technique, considered a purposeful and dynamic tool for clinical risk management. FMEA is noted in five phases that allow a preliminary assessment of a definite process through identification and classification of risk priorities. This study represents the first of a two-phase project where FMEA is applied to HD in the setting of San Feliciano Hospital. The dialysis center performs ~12,000 dialysis sessions per year. The dialysis process is divided into different stages. A total of 31 failure modes were identified in the whole dialysis stages; more than 2/3 of the failure modes were related to the only connecting of the patient to the dialysis machine. The first phase of the study clearly remarked that the most critical step of the dialytic process is represented by the connection between the patient and the machine, as expected. Indeed, in order to have the dialysis set up, an arteriovenous fistula must be surgically created prior to the procedure and it is one of the most important issues in the HD process because of the necessity of a constant revision of it. FMEA application to HD is a useful tool, easy to be implemented and it is likely to nimbly reveal the practical and potential solutions to the critical steps of the procedure.

## Introduction

### Haemodialysis

Haemodialysis (HD) is one of the methods for renal replacement therapy in the management of advanced chronic kidney disease (1).

To purify the blood, HD uses a dialysis machine and a special filter, called a dialyzer. The operating mechanism of the dialysis machine involves the entry of the patient's blood and its purification through an osmosis process. The filter consists of two compartments separated by a membrane, one in which the blood flows and the other one in which the dialysis solution (i.e., dialysate) flows. The dialysate is a special dialysis fluid similar to plasma which flows counter-currently to the blood, so to maximize the concentration gradient of solutes, thus removing urea, creatinine, and other waste products unusually high in the blood; the dialysate, moreover, being constantly replaced, ensures the correct concentration of several solutes in the blood. The membrane, instead, is semipermeable and its very small pores allow the passage of water and solutes, but not that of proteins and blood cells. Moreover, some solutes like Bicarbonate and Calcium, whose concentration in dialysis solution is high, enter the blood section (2).

The main reason why the purifying efficiency of the dialysis machine does not reach that of the healthy kidney is that—apart from the continuous, organic working of healthy kidneys in human bodies—the hemodialytic procedure can take up to 3–6 h and is usually performed three times a week, hence the social, healthcare-associated and economic costs of the procedure itself (3).

The dialytic session involves several stages:

(i) setting up, which consists of controlling the sanitary conditions of the interested hospital wards, testing and dressing the necessary materials, such as the monitor and the equipment of the dialysis machine;
(ii) actual dialysis procedure, formed by patient's evaluation, connection to the extracorporeal circuit, blood circulation circuit activation and its maintenance;
(iii) disconnecting the patient from the extracorporeal circuit.

It clearly appears that dialysis is a highly complex procedure both from a technological and a clinical point of view. Moreover, dialysis patients are frail because of their healthcare condition and because of the not uncommon, several comorbidities that affect them. The complexity of the dialytic procedure often requires the presence of a multi-specialist, multi-disciplinary team (4–6). Therefore, the interaction of these factors makes the dialysis process an important target in clinical risk management (7, 8).

### FMEA and Haemodialysis

Failure Mode and Effect Analysis (FMEA) is a proactive technique, widely used in Human Reliability Analysis (HRA) studies (9–13). HRA's purpose is to examine the activity, process or organizational structure to identify weaknesses and vulnerabilities so that they can be defined and solved. Since its practical use and effective implications, the technique is well-applicable to healthcare practices (14). For instance (15), FMEA has been applied to practical issues such as dosing the right amount of exposition in total body irradiation; it has been used (16) in the diagnostic process, particularly in oncology, where it resulted optimal in the therapeutic decision-making process (17) and, more recently, it showed its potential advantages in surgery (18). In sum, FMEA can be considered a purposeful and dynamic tool for clinical risk management: it is defined as a predictive technique for the identification and classification of risk priorities, which allows a preliminary risks assessment of a process through a qualitative and quantitative analysis aimed at outlining the intervention priorities. The methodological phases of the FMEA are the following:

(i) identification of the target of the analysis;
(ii) identification and description of the activities correlated to the target;
(iii) identification of failure mode(s);
(iv) determination of the risk priority number (RPN) and its analysis;
(v) identification of measures and actions to be implemented as preventive, improvement, and/or corrective acts in order to solve the issue represented by the identified target.

The RPN is the result of the combination of three different assessments as evaluated by a multidisciplinary group; it gathers and accounts for any failure mode or pattern for its severity (S), occurrence (O) and detection (D) ratings. The RPN must be calculated for each recognized cause of failure (19).

In this way, FMEA does not neglect any potential mistake in the execution of the targeted procedure, thus allowing insertion tests and controls, to develop protocols, to prepare countermeasures (20).

Consequently, this study represents the first of a two-phase project where FMEA is applied to HD in a hospital setting. This pilot project is preliminary to the second actualization since in this stage HD was subjected to a descriptive decomposition in order to isolate the several steps which form the dialysis procedure itself with the aim of secluding the most critical stages and approaching them by resolute and/or prophylactic measures. The second phase of the study will evaluate whether the executed implementations have an impact onto the various steps formerly isolated, and the potential magnitude of them.

## Materials and Methods

As far as the FMEA is concerned, a multidisciplinary working group has been set up consisting of eight professionals: nephrologists; nurses; the hospital's clinical risk manager; and experts in risk management from the Sapienza University of Rome.

The working group implemented the FMEA and identified five main stages of the dialysis procedure:

(i) dialysis machine preparation
(ii) connecting the patient to the monitor
(iii) dialysis surveillance
(iv) disconnecting the patient from the monitor
(v) central venous catheter management.

Each of the aforementioned stages was further subdivided into single, practical activities; failure modes and consequent, potential repercussions were isolated as well. The fifth stage was inserted as supernumerary since the relevance of it only when present.

RPN was determined for each failure mode based on a predetermined score, one for each of the three variables included in the calculation (S, O, and D). The categories, the frequencies set up as probability cut-offs, and their related scores are shown in Table 1.

**Table 1:** RPN variables and their labeling and relations to their scores.

**Score**	**Severity**	**Odds**	**Detection**
1	No harm	Very low (1:10,000)	Very high (9:10)
2	Mild harm	Low (1:5,000)	High (7:10)
3	Moderate harm	Moderate (1:200)	Medium (5:10)
4	Severe harm	High (1:100)	Low (2:10)
5	Death	Very high (1:20)	Very low (<1:10)

The contingent presence of any control and/or barrier measure was studied where applicable. According to the results of the RPN score, five categories were set up for the classification of the intervention priorities, and labeled as follows: RPN≥40: “very high”;40 < RPN ≤ 30: “high”;30 < RPN ≤ 20: “medium”; 20 < RPN ≤ 11: “low”; and finally, RPN ≤ 10: “monitoring.” For each of these categories, a color code was assigned, as follows: Red (RPN≥40), Orange (40 < RPN ≤ 30), Yellow (30 < RPN ≤ 20), Green (20 < RPN ≤ 11) and White (RPN ≤ 10).

Thus, a specific master list of priorities was built up summarizing the features taken into consideration in the FMEA.

To include in the FMEA the human variables that may represent unavoidable failure mode(s), clinical features of a sample of the patients requiring dialysis at the San Feliciano Hospital were collected from their medical records after they signed informed consent (21).

The San Feliciano Hospital is an Italian contract clinic in Rome. Among its services, it offers a dialysis center, with two dialysis units for a total of 39 beds, divided into two wards (23 beds in ward A and 16 in ward B). Supplementary beds can be added when needed in particular situations like overcrowding.

In the dialysis center, ~12,000 dialysis sessions are carried out per year, hence the relevance of the chosen hospital for the FMEA application.

The only inclusion criterion, signing informed consent and being ≥18 years old apart, was that to have been subjected to dialysis for more than 6 months. Beyond their anamnestic personal data and clinical records, their potential comorbidities were investigated as well. The Charlson Comorbidity Index (CCI) (22), predicting 10-year survival rate in patients affected by multiple comorbidities, was chosen to gather the pathological conditions of the patients and was used as a proxy to reveal other possible failure modes.

The distribution of the main characteristics of the patients was calculated considering it a normally-distributed sample, thus calculating mean and standard deviation, or its proportion, as appropriate. The statistical software chosen was R, version 4.1.2 (23).

## Results

A total of 79 patients were included in the pilot project as set up by the multidisciplinary group. Their main characteristics are shown in Table 2.

**Table 2:** Main characteristics of the patients under dialysis procedure included in the pilot project at san feliciano hospital.

**Sex (M, %)**	**55 (69.6%)**
**Age (y)**	**68.9 ± 11.7**
**Years under dialysis (y)**	**3.9 ± 4.1**
**CCI**	**5.8 ± 2.1**
**Presence of CVC (%)**	**9 (11%)**
**Presence of prosthesis (%)**	**0 (0)**
**≥3 dialysis sessions per week (%)**	**4 (5%)**

A total of 31 failure modes were identified in the whole dialysis stages; since more than 2/3 of the failure modes were related to the only connecting of the patient to the dialysis machine (stage 2), it was regarded as the most exemplary phase for quantity and variety, and its descriptive results are summarized in Table 3. In this table, the identified Failures Mode(s) are considered as the causes of the potential effects as described in the appropriate column.

**Table 3:** Failure modes and relative RPN in phase 2 of HD process.

**Activity**	**Failure mode(s)**	**Effect(s)**	**RPN**
Patient's clinical evaluation	Missed evaluation	(i) Hypotension (ii) Hypovolemic shock (iii) Cardiac arrest (iv) Inappropriate dialysis	38
	Wrong evaluation		25÷37
Dialysis materials evaluation	Needles verify failure	(i) Process slowdown (ii) Arteriovenous fistula damage	4
	Filter verification failure	Allergic reaction to polysulfone	38
Check patency/suitability of vascular access	AVF failed evaluation	(i) inability to perform dialysis (ii) incomplete and ineffective dialysis (iii) thromboembolism (iv) clot formation in the circuit with loss of hemoglobin (v) bacteremia, sepsis and/or other distant infections	22.5
	AVF wrong evaluation		
	CVC wrong evaluation		18
	CVC wrong management		90
Hands hygiene	Failed	Infection, bacteremia, sepsis	20
	Wrong		
Needle positioning	incorrect positioning/incorrect needle management	(i) extravasation or hematoma for vessel wall injury (ii) FAV closure (iii) incomplete/ineffective dialysis (iv) arterial wall injury with possible pseudo-aneurysm, hemorrhage, ischemia, compartment syndrome, nerve injury	27
Drawing any blood sample	Arteriosus blood sample failure or wrong	Wrong clinical evaluation	15
	Venous blood sample failure or wrong	(i) Wrong clinical evaluation (ii) Bleeding or thrombosis risk	16
Setting dialysis parameters in the monitor	Wrong weight setting	(i) incomplete/ineffective dialysis, (ii) cramps (iii) hypotension, hypovolemic shock	12
	Incorrect weight losing setting		
Needle connection to the machine	Inverted lines	Ineffective dialysis	10
	Incorrect connection closure	blood loss, microbubble formation with need for circuit change (hemoglobin loss)	21
	Defective lines	microbubble formation with need for circuit change (hemoglobin loss)	12
System start with circuit filling	Filter breakage/malfunction	Ineffective dialysis	4.5
	Failure to administer heparin incorrectly	(i) increased risk of bleeding (ii) clot formation in the circuit with loss of hemoglobin	12

The master list directly derived from FMEA, as far as the only second phase is concerned, is shown in Table 4.

**Table 4:** Proposed master list as resulted from the failure modes of the FMEA application to the HD process.

**Activity**	**Failure mode(s)**	**RPN**
Vascular access(es)	Wrong CVC management	90
	Wrong/failed AVF evaluation	22.5
	Wrong CVC evaluation	18
Clinical evaluation	Wrong/failed evaluation	25÷37
Dialysis materials evaluation	Needles verify failure	36
	Filter verification failure	4
System start with circuit filling	Wrong heparin administration	35
	Failed heparin administration	12
	Filter breakage/malfunction	4.5
Needle positioning	Incorrect positioning/incorrect needle management	27
Needle connection to the machine	Incorrect connection closure	21
	Defective lines	12
	Inverted lines	10
Hands hygiene	Wring/failed hands hygiene	20
Drawing any blood sample	Arteriosus blood sample failure or wrong	16
	Venous blood sample failure or wrong	15
Setting dialysis parameters in the monitor	Wrong weight or weight losing setting	12

## Discussion

The first phase of the study clearly remarked that the most critical step of the dialytic process is represented by the connection between the patient and the machine, as expected (24, 25). Contrary to what other Authors found, miscommunication difficulties did not represent significant issues in our case (24, 26). Indeed, in order to have the dialysis set up, an arteriovenous fistula must be surgically created prior to the procedure and it is one of the many issues in the HD process because of the necessity of a constant revision of it (4).

The presence of a CVC is the only red-flagged element in the whole considered stage of HD; in particular, its wrong management has the highest RPN value, while a wrong evaluation of it seemed not to affect as much the procedure, as remarked by others (24, 25). Despite the difference between evaluation and management could actually be idle, what the results of the FMEA highlighted is that when patients carry a CVC and they have to undergo an HD session, they should be regarded as high-risk patients when compared to the other dialyzed. CVCs in fact represent the second discontinuation of the barrier given by skin—being the AVF the first one. This medical device, although useful for liquids and drugs administration, enhances the risks of suffering from infections of any kind, especially those known as healthcare-acquired, thus boosting the risk of incurring in sepsis and death (26, 27). When the variable represented by CVCs is erased from the model, HD's more critical stage is the clinical evaluation. This implicates that an adequate and up-to-date education of the staff, both nurses and physicians, should be enough to guarantee a successful and eventless HD session. In this sense, the correct method of hand sanitization should be one of the first healthcare procedures to be learned by the staff, without considering it irrelevant since the diffusion of its implementation due to the COVID pandemic (20, 28).

Needles management and their correct use is another relevant action liable for what concerns failure modes. Needles prominence is best explained by the high RPN related to the administration of heparin, although it is uncommon: since patients requiring HD are necessarily submitted to anticoagulation drugs (28), if important arteries are damaged during needle insertion, emergency protocols, and blood transfusions must be carried out. Mainly and more frequently, poor needle management makes dialysis inefficient and/or ineffective. Moreover, needles represent another risky device for what concerns healthcare-acquired infections as well as work-related accidents, meaning that potential harm to the staff must be considered, too (29, 30).

What aforementioned openly shows that, although HD is a well-known and established procedure, it can be extremely risky for patients and for hospital staff as well; however, since scarce the critical points appear to be, the prophylactic interventions should be easily carried out in hospital settings.

As far as the patient's sample is considered, several are the implication of it for the second phase of our project. First, we should include patients with some kind of prosthesis, thus adding the presence of it to the FMEA process, to highlight any other critical step. Secondly, female patients should be more thoroughly evaluated, so that women's peculiarities—such as hormonal diversity, compliances differences and any other issue belonging to the so-called gender medicine—are not forgotten and/or neglected (31).

The length of the dialysis treatment for the analyzed sample resulted in being sufficient to consider patients as clinically stable so that their comorbidities could not represent a bias in the evaluation of the FMEA. On the other hand, the not insignificant value of the CCI will undergo a further and deeper analysis in the second phase of the study. Indeed, the 10-year survival rate associated with a CCI value of almost 6 points is equal to 2%. General Italian population coeval to the analyzed sample has a probability of surviving (32) for almost 20 years equal to 90%: this easy comparison emphasizes how the dialytic population is intrinsically prone to get serious complications even from the medical procedures considered as the safest and the most efficient ones.

Nonetheless, at the same time, age could be the more treacherous variable in this sense, because of the comorbidities related to the aging process, along with the condition of frailty in the elderly incur as years go by.

The application of the FMEA process showed that specific strategies for each failure mode, as far as improvement activities from a clinical, organizational, and training point of view, must be listed as well, so that a Risk Management Plan can be built up for HD. In fact, FMEA guarantees an adequate analysis of risk priorities as aforementioned, other than providing a series of relevant information in the selected care setting. Moreover, FMEA results clearly point out where to best allocate the financial resources, in order to prevent risks and improve organizational conditions more effectively.

In this way, the multidisciplinary working group shall enact all the possible directions in order to have the staff and patients informed about the risks and, especially, how to avoid them, with a prophylactic aim. It should be underlined that, recently, legislation changes in Italy have made medical malpractice suits easier to be submitted, hence the huge amount of litigations and costs related to the so-called defensive medicine (33, 34). Furthermore, any tool aimed at anticipating potential claims should be thoroughly considered and implemented, when adequate (35, 36).

## Limitations

FMEA application to HD is a useful tool, easy to be implemented and it is likely to nimbly reveal the practical and potential solutions to the critical steps of the procedure. The evaluation of the solutions as identified by the current analysis will be tested, as already mentioned above, in the second phase of the study. However, being dialysis a procedure known and used since the second half of the XX centuries, it is very likely that FMEA results will not be dissimilar if other similar techniques should be implemented; at least, the debate could be about the proposed resolutions and operative arrangements (37, 38).

Another issue is that of applicability: FMEA is a method well-known in other specialties; however, its actual efficacy concerning dialysis has not been recognized yet. For this main reason, further studies investigating the application of this pilot study to broader research will be enlightening.

## Data Availability Statement

The raw data supporting the conclusions of this article will be made available by the authors, without undue reservation.

## Ethics Statement

Ethical review and approval was not required for the study on human participants in accordance with the local legislation and institutional requirements. Written informed consent for participation was not required for this study in accordance with the national legislation and the institutional requirements.

## Author Contributions

RLR, VF, and MF: conceptualization. NDF: methodology. RV: validation. GP: formal analysis. GC: data curation. FM and PF: writing—original draft preparation. VF and MF: writing—review and editing. PF and RLR: supervision. All authors have read and agreed to the to the published version of the manuscript and contributed to the drafting and critical revision of the work.

## Conflict of Interest

The authors declare that the research was conducted in the absence of any commercial or financial relationships that could be construed as a potential conflict of interest.

## Publisher's Note

All claims expressed in this article are solely those of the authors and do not necessarily represent those of their affiliated organizations, or those of the publisher, the editors and the reviewers. Any product that may be evaluated in this article, or claim that may be made by its manufacturer, is not guaranteed or endorsed by the publisher.
